# Adenovirus-mediated hypoxia-targeting cytosine deaminase gene therapy enhances radiotherapy in tumour xenografts

**DOI:** 10.1038/sj.bjc.6603812

**Published:** 2007-05-22

**Authors:** J Liu, H Harada, M Ogura, T Shibata, M Hiraoka

**Affiliations:** 1 Department of Radiation Oncology and Image-Applied Therapy, Kyoto University Graduate School of Medicine, 54 Shogoin Kawahara-cho, Sakyo-ku, Kyoto 606-8507, Japan; 2 Nano-Medicine Merger Education Unit, Kyoto University, 54 Shogoin Kawahara-cho, Sakyo-ku, Kyoto 606-8507, Japan

**Keywords:** tumour hypoxia, hypoxia-response element (HRE), hypoxia-inducible factor-1 (HIF-1), adenovirus, gene therapy, radiotherapy

## Abstract

Hypoxia is closely associated with the radioresistance of tumours; therefore, targeting hypoxic areas is very important for cancer therapy. The aim of this study is to establish such a targeting strategy by applying a bacterial cytosine deaminase (BCD)/5-fluorocytosine (5-FC) gene therapy system and to examine whether the strategy enhances the efficacy of radiotherapy in a tumour xenograft. The hypoxia-responsive promoter 5HREp, in which five copies of the hypoxia-response element (HRE) enhance transcription from a cytomegalovirus minimal promoter, was employed to induce the expression of BCD under hypoxic conditions. The adenoviral vector Ad/5HREp-BCD, encoding the gene *5HREp-BCD*, robustly induced BCD expression under hypoxic conditions and this led to significant cytotoxicity in combination with 5-FC *in vitro*. Intratumoral Ad/5HREp-BCD administration resulted in the expression of BCD at the border between normoxic and necrotic regions. The BCD/5-FC gene therapy enhanced the therapeutic effects of both single (12.5 Gy) and fractionated (3 Gy × 5 days) radiotherapy with few side effects and significantly increased tumour growth doubling time by up to 2.4-fold (*P*<0.01) and 2.5-fold (*P*<0.05), respectively. All of these results suggest that the present BCD/5-FC gene therapy has the ability to specifically target hypoxic tumour cells and significantly improves the control of tumour growth after radiotherapy.

The exponential proliferation of cancer cells and the resultant distance that develops between nutritive blood vessels and some tumour cells result in an imbalance in the supply and consumption of oxygen in solid tumours. Such disequilibrium is a major causative factor of tumour hypoxia, a characteristic microenvironment in locally advanced solid tumours (Thomlinson and Gray, 1955; Vaupel *et al*, 1989). The hypoxia is closely associated with malignant phenotypes (Graeber *et al*, 1996), metastasis (Rofstad, 2000), invasion (Pennacchietti *et al*, 2003), and angiogenesis (Harris, 2002). The hypoxic fraction correlates to the resistance to chemotherapy (Teicher, 1994) and radiotherapy (Thomlinson and Gray, 1955; Brown and Wilson, 2004). Therefore, not only has tumour hypoxia been considered an adverse prognostic indicator, but also, a hypoxia-targeting strategy is becoming increasingly important to overcome these problems (Teicher, 1994; Harris, 2002; Brown and Wilson, 2004).

Under hypoxic conditions, hypoxia-inducible factor-1 (HIF-1) plays a pivotal role in inducing the expression of various genes (Semenza, 2001). Hypoxia-inducible factor-1 is a heterodimeric transcription factor composed of an *α*-subunit (HIF-1*α*) and a *β*-subunit (HIF-1*β*) (Wang *et al*, 1995). The expression of HIF-1*α* is regulated in an oxygen-dependent manner mainly at the post-translational level and is responsible for the regulation of HIF-1's activity (Kallio *et al*, 1997). Proline residues in the oxygen-dependent degradation domain of HIF-1*α* protein are hydroxylated under normoxic conditions (Jaakkola *et al*, 2001). The modified HIF-1*α* protein is ubiquitinated by E3 ubiquitin–protein ligases containing the von Hippel–Lindau tumour suppressor protein (pVHL) and rapidly degraded by the 26S proteasome (Jaakkola *et al*, 2001). On the other hand, the rate at which proline was hydroxylated decreased under hypoxic conditions, resulting in a reduced rate of ubiquitination and subsequent degradation (Jaakkola *et al*, 2001). The stabilised HIF-1*α* interacts with the constitutively expressed HIF-1*β* protein and induces the gene expression of erythropoietin (Wang and Semenza, 1993), VEGF (Forsythe *et al*, 1996), and others (Semenza, 2001). The induction is triggered by the interaction of HIF-1 with its cognate DNA recognition site, the hypoxia-response element (HRE) (Norris and Millhorn, 1995; Forsythe *et al*, 1996). An increased level of HIF-1*α* in the tumour and the resultant upregulation of HIF-1 activity as well as tumour hypoxia have been associated with tumour malignancy, aggressive tumour growth, tumour radioresistance and a poor prognosis (Powis and Kirkpatrick, 2004).

Extensive efforts have focused on the development of biological approaches to deal with tumour hypoxia (Semenza, 2003; Brown and Wilson, 2004). One of the most striking advances is the development of artificial hypoxia-responsive promoters (Greco *et al*, 2000), in which the HRE(s) has been utilised as a transcriptional enhancer. Many groups have reported that a tandem repeat of HREs enhances gene expression under hypoxic conditions (Greco *et al*, 2000). Above all, the 5HRE promoter (5HREp), in which five copies of the HRE enhance transcription from a cytomegalovirus (CMV) minimal promoter, enhances gene expression more than 500-fold under hypoxic conditions *in vitro* (Shibata *et al*, 1998, 2000; Greco *et al*, 2000). Optical imaging of tumour hypoxia by using the 5HREp-luciferase gene and the *5HREp-*green fluorescent protein (GFP) gene has proved the potential of the promoter *in vivo* as well as *in vitro* (Vordermark *et al*, 2001; Harada *et al*, 2005; Liu *et al*, 2005). Hypoxia-specific targeting was also accomplished *in vivo*, when cytotoxic genes or therapeutic genes, such as for apoptotic factors or prodrug-activating enzymes, were inserted downstream of the hypoxia-responsive promoters (Greco *et al*, 2000; Koshikawa *et al*, 2000; Patterson *et al*, 2002; Shibata *et al*, 2002; Binley *et al*, 2003; Ogura *et al*, 2005). However, all of these *in vivo* experiments were conducted using stable transfectants with each hypoxia-responsive gene. In other words, no one has examined whether 5HREp would function in a *trans*-acting gene therapy strategy.

In the present study, we utilised 5HREp (Shibata *et al*, 2000) and a prodrug-activating gene, bacterial cytosine deaminase (BCD) (Mullen *et al*, 1992; Miller *et al*, 2002), and successfully established an adenovirus-mediated gene therapy strategy for tumour hypoxia. We used this strategy to determine whether the specific targeting of tumour hypoxia by gene therapy improves the efficacy of radiotherapy in a tumour xenograft.

## MATERIALS AND METHODS

### Cell culture

The human cervical epithelial adenocarcinoma cell line HeLa and the human pancreatic carcinoma cell line MIA PaCa-2 were maintained in Dulbecco's modified Eagle's medium with 10% fetal bovine serum. The human pancreatic carcinoma cell line CFPAC-1 was maintained in Iscove's modified Dulbecco's medium (IMDM) with 10% fetal bovine serum. The human colon carcinoma cell lines WiDr and HT29 were maintained in RPMI-1640 medium with 10% fetal bovine serum. All cell lines were purchased from American Type Culture Collection. For normoxic incubation, the cells were incubated in a well-humidified incubator with 5% CO_2_ and 95% air at 37°C.

### Plasmid DNA

To construct the plasmid pEF/BCD, which constitutively expresses a BCD protein fused to a myc epitope tag under the control of the EF-1*α* promoter, a DNA fragment for the *Escherichia coli codA* gene, which encodes the enzyme cytosine deaminase, was amplified by PCR and inserted between *Nco*I and *Not*I recognition sites of the vector pEF/myc/cyto (Invitrogen, Carlsbad, CA, USA). To construct the plasmid p5HRE/BCD, which induces the expression of the BCD-myc fusion protein under hypoxic conditions, a DNA fragment for 5HREp was obtained by digestion with *Kpn*I and *Nco*I from the vector, 5HRE/hCMVmp (Shibata *et al*, 2000), inserted between *Kpn*I and *Nco*I recognition sites of pEF/BCD, and substituted for the constitutive EF-1*α* promoter.

### Stable transfectants

To establish stable transfectants, HeLa/EFp-BCD and HeLa/5HREp-BCD, HeLa cells (1 × 10^5^) were transfected with pEF/BCD and p5HRE/BCD, respectively, by a modified calcium–phosphate method (Chen and Okayama, 1987, 1988). Twenty-four hours after the transfection, the culture medium was refreshed with selection medium containing 5 *μ*g ml^−1^ of blasticidine for HeLa/EFp-BCD cells or 400 *μ*g ml^−1^ of G418 for HeLa/5HREp-BCD cells. Each antibiotic-resistant cell culture was directly used for both the Western blot analysis and the *in vitro* cell proliferation assay.

### Construction, amplification, and infection of the adenovirus

To construct cosmid vectors, pAxcw/EFp-BCD and pAxcw/5HREp-BCD, DNA fragments for *EFp-BCD* and *5HREp-BCD* were prepared from pEF/BCD and p5HRE/BCD, respectively, by digestion with *Kpn*I and *Bam*HI, blunted and inserted into the *Swa*I recognition site of the cosmid vector pAxcw (TaKaRa, Tokyo, Japan). The recombinant adenoviruses, Ad/EFp-BCD and Ad/5HREp-BCD, were generated by COS–TPC methods (Miyake *et al*, 1996) using an adenovirus expression kit according to the manufacturer's instructions (TaKaRa). For large-scale preparations, the adenoviruses were amplified in a transformed human embryonic kidney cell line, 293, and purified by two steps of caesium chloride density centrifugation. Viral titers were measured in a limiting-dilution bioassay using 293 cells. Cells (1 × 10^5^ cells per dish) were seeded onto a 60 mm dish and treated with Ad/EFp-BCD or Ad/5HREp-BCD for 1 h. Then, the adenovirus-containing medium was replaced with one without the virus.

### Western blot analysis

The stable transfectants and the virus-infected cells were seeded in 60 mm glass dishes (2 × 10^5^ cells per dish), put into pre-warmed aluminium chambers, and flushed with hypoxic gas (95% N_2_, 5% CO_2_) for 30 min. Then, tightly sealed chambers were incubated at 37°C for 16 h for the hypoxic treatment. The cells were harvested in RIPA lysis buffer (10% SDS, 2 M Tris-HCl, pH 7.5, and 1% Triton X) supplemented with a protease inhibitor, Mini complete (Roche, Basel, Switzerland). The lysates were sheared using a syringe with a 23-gauge needle, and the protein concentration was determined using the DC Protein assay kit (Bio-Rad). Twenty micrograms of total protein was electrophoresed on a 12% SDS polyacrylamide gel, transferred onto PVDF membrane (GE Healthcare Bio-Sciences Corp., Piscataway, NJ, USA) and blocked with 5% non-fat milk in Tris-buffered saline. The BCD protein fused to the myc epitope tag was detected with monoclonal anti myc-tag antibody (Cell Signaling Technology Inc., Danvers, MA, USA) and anti mouse IgG horseradish peroxidase-linked whole antibody (GE Healthcare Bio-Sciences Corp.) using an ECL-PLUS system (GE Healthcare Bio-Sciences Corp.) according to the manufacturer's instructions.

### *In vitro* cell proliferation assay

The stable transfectants and the virus-infected cells were seeded in triplicate into 96-well plates (1 × 10^3^ cells per well) and incubated with various concentrations of 5-fluorocytosine (5-FC) (Sigma Chemical Co., St Louis, MO, USA) for 24 h under normoxic or hypoxic conditions. For the hypoxic treatment (<0.02% of oxygen), the cells were treated in a hypoxic chamber, BACTRON-II (Sheldon Manufacturing Inc., Cornelius, OR, USA). The cells were additionally incubated under normoxic conditions for 24 h. The culture medium was then changed to one without 5-FC, and the cells were cultured for 48 h under the normoxic conditions. Cell growth inhibition was quantified by colorimetric assay using a CellTiter 96 AQueous One Solution Cell Proliferation Assay Kit (Promega, Madison, WI, USA) according to the manufacturer's instructions.

### Tumour-bearing mice

A suspension of HeLa cells (2 × 10^6^ cells/100 *μ*l of PBS) was subcutaneously inoculated into the right hind leg of a 6-week-old nu/nu BALB/c mice (Charles River, Tokyo, Japan).

### Immunohistochemical analysis

The adenovirus Ad/EFp-BCD or Ad/5HREp-BCD was intratumorally injected into the HeLa tumour xenografts, when the xenografts developed to approximately 150–200 mm^3^. Four days later, pimonidazole hydrochloride (Natural Pharmacia International Inc., Belmont, MA, USA) was intraperitoneally (i.p.) injected into the tumour-bearing mice (60 mg kg^−1^). Ninety minutes later, the tumours were surgically excised, immediately fixed in 10% formalin neutral buffer solution (pH=7.4; Wako Pure Chemical Industries Inc., Osaka, Japan), and embedded in paraffin. To detect pimonidazole and BCD-myc, paraffin-embedded sections were treated with anti-pimonidazole (Natural Pharmacia International Inc.) and anti-*c*-myc antibody (Santa Cruz, CA, USA), respectively, and stained using an indirect immunoperoxidase detection method (DakoCytomation, Carpinteria, CA, USA), according to the manufacturer's instructions. Counterstaining with haematoxylin was also carried out. Paraffin-embedded serial sections were also stained with haematoxylin–eosin (HE).

### Radiation conditions

The tumour-bearing mice were irradiated at 1.468 Gy min^−1^ with an X-ray irradiation machine (SHIMADZU, Kyoto, Japan). All the tumour-bearing mice were restrained and shielded with a specially designed lead apparatus that allowed local irradiation to the tumour on the right hind leg.

### Growth delay assays

When the tumour xenografts developed to approximately 150–200 mm^3^, the tumour-bearing mice were randomly divided into five treatment groups: (1) a sham-treated group, (2) an Ad & 5-FC group, (3) an ionising radiation (IR) group, (4) an IR & 5-FC group and (5) an Ad & 5-FC & IR group. In the single irradiation experiment, 2 × 10^9^ PFU of adenovirus was intratumorally injected into the mice of the Ad & 5-FC and Ad & 5-FC & IR groups on day 0. 5-FC (500 mg kg^−1^) was i.p. injected into the mice of the Ad & 5-FC, IR & 5-FC, and Ad & 5-FC & IR groups on both day 1 and day 2. Irradiation (12.5 Gy) was applied to the mice of the IR, IR & 5-FC, and Ad & 5-FC & IR groups 12 h after the injection of 5-FC on day 1. In the fractionated irradiation experiment, the adenovirus was intratumorally injected into the mice of the Ad & 5-FC and Ad & 5-FC & IR groups on day 0. 5-Fluorocytosine was administered daily from day 1 to day 5 to the mice of the Ad & 5-FC, IR & 5-FC, and Ad & 5-FC & IR groups. Irradiation was applied 12 h after the injection of 5-FC daily from day 1 to day 5 to the mice of the IR, IR & 5-FC, and Ad & 5-FC & IR groups (3 Gy × 5 days). For the negative control, PBS was injected instead of the adenovirus and the 5-FC. The tumour size was measured with calipers, and the tumour volume was calculated as 0.5*LW*^2^.

### Statistical analysis

The statistical significance of differences was determined using the Student's *t*-test (*P*<0.05).

## RESULTS

### Establishment of a hypoxia-dependent prodrug-activating system

**Figure 1:**
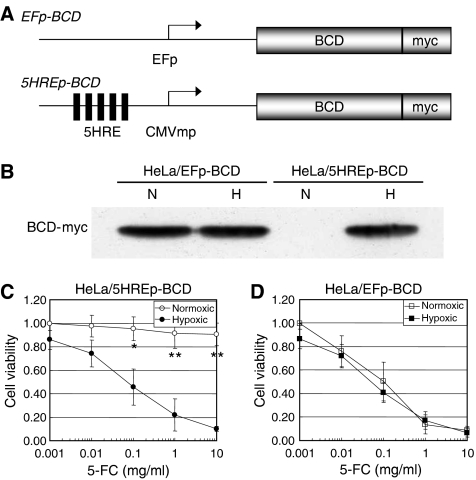
Hypoxia-responsive BCD expression and 5-FC sensitivity. (**A**) Schematic diagrams of the *EFp-BCD* gene constitutively expressing the BCD (top) and the *5HREp-BCD* gene hypoxia-dependently expressing the BCD (bottom). The BCD coding gene was fused to the myc epitope in frame. (**B**) Western blot analysis of BCD-myc expression in HeLa/EFp-BCD cells or HeLa/5HREp-BCD cells under normoxic (N) or hypoxic (H) conditions. (**C** and **D**) Cell proliferation assay of HeLa/5HREp-BCD cells (**C**) and HeLa/EFp-BCD cells (**D**). The cells were treated with various concentrations of 5-FC under normoxic (open) or hypoxic (solid) conditions. Cell viability was calculated as the ratio of the absorbance value in each of the conditions against that in medium with 0.001 mg ml^−1^ of 5-FC under normoxic conditions. Results are the mean±s.d. (*n*=3). ^*^P<0.05. ^**^P<0.01.

To establish a hypoxia-targeting gene therapy strategy, we first constructed a plasmid, p5HRE/BCD, encoding the *5HREp-BCD* gene (Figure 1A). Shibata *et al* (2000) employed 5HREp to induce the therapeutic gene expression specifically under hypoxic conditions. The *BCD* gene was used as the prodrug-activating gene, because the intratumoral production of 5-fluorouracil by the BCD/5-FC system is effective for cancer therapy (Mullen *et al*, 1992; Miller *et al*, 2002). HeLa cells were stably transfected with p5HRE/BCD and the hypoxia dependency of the BCD expression was examined by Western blot analysis (Figure 1B). The stable transfectant, HeLa/5HREp-BCD, expressed the BCD protein only under hypoxic conditions, while the HeLa/EFp-BCD cells, which had been expected to express constitutively the protein, indeed expressed BCD regardless of the conditions. We next examined whether the hypoxia-dependent BCD expression led to the hypoxia-specific cytotoxicity. The HeLa/5HREp-BCD cells were treated with various concentrations of 5-FC under normoxic or hypoxic conditions, and the growth inhibitory effects were assessed by MTS assay. Significant growth inhibition was observed only under hypoxic conditions (*P*<0.05 with 0.1 mg ml^−1^ of 5-FC, *P*<0.01 with 1 and 10 mg ml^−1^ of 5FC). There was no significant inhibition observed under normoxic conditions (Figure 1C). On the other hand, HeLa/EFp-BCD cells showed hypoxia-independent sensitivity to the 5-FC treatment (Figure 1D). Thus, we confirmed that the 5HREp-dependent BCD/5-FC strategy led to the hypoxia-specific cytotoxicity.

### Ad/5HREp-BCD-mediated cytotoxicity under hypoxic conditions

**Figure 2:**
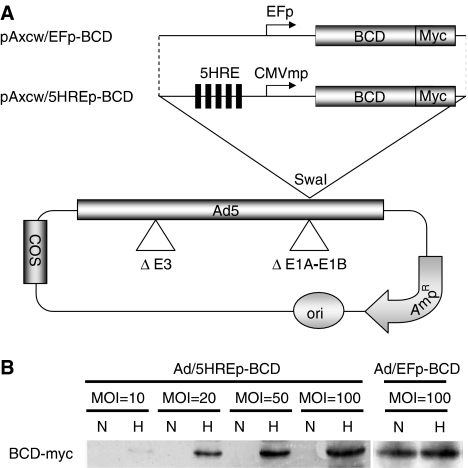
Adenovirus-mediated BCD expression under hypoxic conditions. (**A**) Schematic diagrams of the cosmid vectors, pAxcw/EFp-BCD (top) and pAxcw/5HREp-BCD (bottom), encoding *EFp-BCD* and *5HREp-BC*D, respectively. Ori=replication origin; Amp^R^=ampicillin-resistance gene; COS=cos (phage *λ* sequences) region (**B**) Western blot analysis of BCD-myc expression by using anti myc-tag antibody. HeLa cells were infected with Ad/EFp-BCD or Ad/5HREp-BCD at the indicated MOI, and exposed to normoxic (N) or hypoxic (H) conditions.

We decided to use an adenovirus to transduce the *5HREp-BCD* gene into tumour cells *in vivo*, because the adenovirus is one of the most effective vectors with which to accomplish *trans*-gene expression. We constructed a cosmid vector, pAxcw/5HREp-BCD (Figure 2A), and obtained the adenovirus, Ad/5HREp-BCD, by the COS–TPC methods (Miyake *et al*, 1996). To examine whether Ad/5HREp-BCD showed hypoxia-dependent BCD expression, we performed a Western blot analysis (Figure 2B). HeLa cells were infected with Ad/5HREp-BCD at a MOI of 10–100 and cultured under normoxic or hypoxic conditions. The BCD protein was expressed only under hypoxic conditions. The amount of BCD protein expressed and the ratio of the expression under hypoxia to that under normoxia increased with the increase in the MOI. On the other hand, HeLa cells infected with Ad/EFp-BCD constitutively expressed BCD protein regardless of oxygen conditions (Figure 2B).

**Figure 3:**
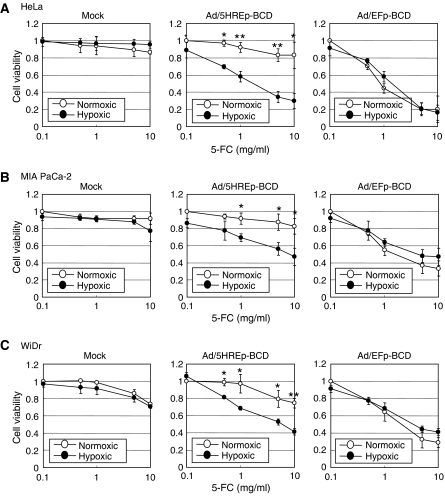
Ad/5HREp-BCD-mediated cytotoxicity. (**A**) HeLa, (**B**) MIA PaCa-2, and (**C**) WiDr cells were infected with the adenovirus, Ad/EFp-BCD or Ad/5HREp-BCD, and treated with various concentrations of 5-FC under normoxic (open) or hypoxic (solid) conditions. Cell viability was calculated as the ratio of the absorbance value under each of the conditions against that in medium with 0.1 mg ml^−1^ of 5-FC under normoxic conditions. The same experiment was conducted with mock infection. Results are the mean±s.d. (*n*=3). ^*^P<0.05. ^**^P<0.01.

We next evaluated the hypoxia dependency and the therapeutic efficacy of the Ad/5HREp-BCD-mediated strategy *in vitro*. The virus-infected HeLa cells were exposed to various concentrations of 5-FC under normoxic or hypoxic conditions, and the growth inhibitory effect was examined by MTS assay (Figure 3A). The cell proliferation was significantly inhibited under hypoxic conditions compared to normoxic conditions when the cells were treated with Ad/5HREp-BCD (MOI=100) and the higher concentration of 5-FC. Likewise, MIA PaCa-2 and WiDr cells showed hypoxia-dependent sensitivity to the adenovirus-mediated BCD/5-FC treatment (Figure 3B and C). On the other hand, proliferation was inhibited under both normoxic and hypoxic conditions, when the cells were infected with Ad/EFp-BCD (MOI=100). All of the *in vitro* experiments clearly indicate that our system functioned as we desired.

### Hypoxia-specific BCD expression after intratumoral Ad/5HREp-BCD injection

**Figure 4:**
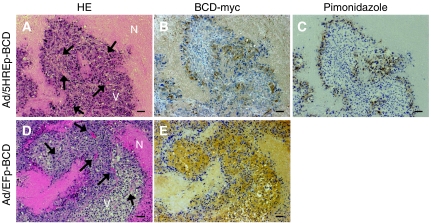
Immunohistochemical analysis of BCD expression in virus-injected tumour xenografts. The tumour xenograft of HeLa cells was intratumorally injected with Ad/5HREp-BCD (**A–C**) or Ad/EFp-BCD (**D** and **E**). Serial sections of the xenograft were subjected to HE staining (**A** and **D**), and to immunohistochemical analysis with anti-*c*-myc antibody for the detection of BCD-myc (**B** and **E**), and with anti-pimonidazole antibody (**C**). Bar=100 *μ*m. N=necrotic tumour tissue; V=well-oxygenated viable tumour tissue; arrow=blood vessel.

We examined whether Ad/5HREp-BCD induces the expression of BCD in hypoxic regions of the tumour xenograft. The virus (1 × 10^9^ pfu) was intratumorally injected into HeLa tumour xenografts, and the regions expressing BCD were compared to those stained with a marker of hypoxia, pimonidazole (Durand and Raleigh, 1998). The immunohistochemical analysis showed that the hypoxic cells stained with pimonidazole were located about 100 *μ*m from a tumour blood vessel, and a robust expression of BCD was also observed there (Figure 4A–C). On the other hand, remarkable BCD expression was observed in well-oxygenated viable regions after intratumoral injection of Ad/EFp-BCD (Figure 4D and E). These results suggest that the *trans*-gene expression of BCD in hypoxic tumour cells can be achieved by the intratumoral administration of the adenovirus Ad/5HREp-BCD.

### Improvement of radiotherapy by Ad/5HREp-BCD-mediated gene therapy

**Figure 5:**
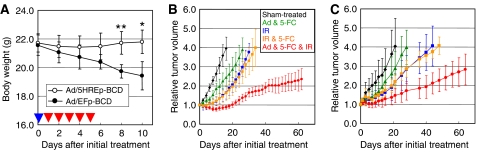
Synergistic antitumour effect of a combination of gene therapy with IR treatment. (**A**) Ad/EFp-BCD or Ad/5HREp-BCD was intratumorally injected into tumor-bearing mice on day 0 (blue arrow head), and 5-FC was administered daily from day 1 to day 5 (red arrow head). Body weight of the tumour-bearing mice was measured during and after the treatment. The results are the mean of six independent mice±s.d. ^*^P<0.05. ^**^P<0.01. (**B** and **C**) Tumour-bearing mice in the Ad & 5-FC group and Ad & 5-FC & IR group were administered Ad/5HREp-BCD. Those in the Ad & 5-FC group, IR & 5-FC group, and Ad & 5-FC & IR group were administered 5-FC. The tumour xenografts in the IR group, IR & 5-FC group, and Ad & 5-FC & IR group were locally exposed to irradiation with a single dose of 12.5 Gy (**B**) or daily dose of 3 Gy for 5 days (**C**). (See Materials and methods for details.) Tumour volume was measured with calipers and calculated as 0.5 *L W*^2^. Relative tumour volume indicates the ratio of the tumour volume on each day to the corresponding volume on day 0. The results are the mean of six independent tumours±s.d.

The *in vitro* cell proliferation assay (Figure 3) and the immunohistochemical analysis (Figure 4) led us to expect a hypoxia-specific therapeutic effect of the Ad/5HREp-BCD, and 5-FC gene therapy. Actually, we confirmed an advantage of 5HREp concerning side effects on normal tissues. The Ad/5HREp-BCD/5-FC gene therapy caused no obvious side effects, while the Ad/EFp-BCD/5-FC gene therapy, despite the local administration, caused significant weight loss (Figure 5A) and severe diarrhea (data not shown). This result indicates that our system functioned, as we desired.

We next examined whether the combination of the Ad/5HREp-BCD/5-FC gene therapy with radiotherapy produced a synergistic antitumour effect. We treated HeLa tumour xenografts with the gene therapy (Ad & 5-FC) and/or radiotherapy (IR) and carried out growth delay assays (Figure 5B). We intentionally chose a relatively low dose of Ad & 5-FC, which had minimal effects on the tumour growth rate. Therefore, tumour growth after the gene therapy alone (Ad & 5-FC group) was not significantly suppressed compared to that after sham-treatment (sham-treated group). On the other hand, combined with IR (Ad & 5-FC & IR group), the gene therapy strikingly suppressed tumour growth as compared to radiotherapy alone (IR group). The period taken for tumour growth to increase two-fold from the initial volume (tumour growth doubling time, TGDT) more clearly shows the therapeutic effect of the treatment (Table 1). The TGDT after gene therapy alone (Ad & 5-FC group) was 13.2±5.6 days, which is not significantly longer than that after sham-treatment (8.2±3.1 days; *P*=0.144). On the other hand, the combination of gene therapy with radiotherapy (Ad & 5-FC & IR) prolonged the TGDT to 47.2±16.8 days, which was about 2.4-fold longer than that after radiotherapy alone (IR group; 19.4±4.8 days; *P*<0.01). Thus, we confirmed that the adenovirus-mediated and hypoxia-targeting gene therapy significantly enhances the effect of radiotherapy.

Similar results were obtained in the experiment using the fractionated irradiation (3 Gy × 5 fractions: Figure 5C). The TGDT after gene therapy alone (Ad & 5-FC group) was 13.0±4.4 days, which is not significantly longer than that after sham treatment (9.8±5.8 days; *P*=0.148). On the other hand, the TGDT after the fractionated radiotherapy (IR) was 17.0±3.7 days, which was significantly delayed by the combination with the gene therapy (Ad & 5-FC & IR group) to 43.3±23.8 days (Table 1; *P*<0.05). These results further strengthen the conclusion that hypoxia targeting by gene therapy improves the efficacy of radiotherapy.

## DISCUSSION

In the present study, we established a hypoxia-targeting strategy by applying a BCD/5-FC gene therapy system and examined whether the strategy enhances the efficacy of radiotherapy in a tumour xenograft.

Because tumour hypoxia has been recognised as a tumour-specific microenvironment, recent research has tried to exploit it as a crucial target for cancer therapy (Harris, 2002; Semenza, 2003; Brown and Wilson, 2004). In this regard, the hypoxia-specific gene therapy strategy has been focused on, and artificial hypoxia-responsive promoters have been developed using various kinds of HREs, such as murine PGK-1 HRE, human erythropoietin HRE, and human VEGF HRE (Greco *et al*, 2000). Above all, 5HREp showed the best hypoxia-responsiveness and exhibited a more than 500-fold increase in luciferase activity in response to hypoxic stimuli (Shibata *et al*, 2000). The absolute level of luciferase activity from 5HREp under the hypoxic conditions reached the same level as that from the CMV-driven promoter under normoxic conditions (Shibata *et al*, 2000). Consistent with these previous reports, the expression of BCD was robustly induced under hypoxic conditions in our plasmid based and adenovirus-based Western blot analysis. This induction actually led to significant hypoxia-dependent cytotoxicity in our cell proliferation assay.

The sensitivity of each cell line to the Ad/5HREp-BCD/5-FC treatment varied in the present cell proliferation assay (Figure 3; compare the viability of each cell line at MOI=100). Among the cell lines tested, HeLa cells exhibited the highest hypoxia dependency concerning sensitivity to the treatment. On the other hand, a human colon carcinoma cell line, HT29, and a human pancreatic carcinoma cell line, CFPAC-1, showed little therapeutic efficacy (Supplementary Figure S1A and B). We hypothesised that this variability might be caused by the difference in the efficiency of adenoviral infection in each cell line, because it was reported that cells showed different infection efficiencies and CFPAC-1 cells had the lowest transduction efficiency among cells tested (Bouvet *et al*, 1998). We performed a chemiluminescent *β*-gal assay to analyse the efficiency of the adenoviral infection and confirmed that HeLa cells showed the highest, and HT29 and CFPAC-1 cells, a much lower, infection efficiency (Supplementary Table S1). Moreover, when we transfected HT29 and CFPAC-1 cells with p5HRE/DsRed2 plasmid (not an adenovirus), we confirmed the presence of hypoxia-dependent red fluorescence, indicating that the 5HREp works in these cells (Supplementary Figure S2). Therefore, we concluded that the low infection efficacy of the adenovirus was responsible for the weak therapeutic efficacy in HT29 and CFPAC-1 cells. These results indicate that, although hypoxia is a common feature of solid tumours, our hypoxia-targeting system cannot target all tumour hypoxia without an excellent vector.

To measure the damage to normal tissue after hypoxia-targeting treatment, Binley *et al* (2003) applied a hypoxia-responsive thymidine kinase/ganciclovir (TK/GCV) strategy and evaluated the activity of lactate dehydrogenase (LDH) as an indicator of liver dysfunction. Hypoxia-dependent TK expression and GCV treatment caused no irregularity in LDH levels. On the other hand, constitutive TK expression from a CMV promoter and GCV treatment significantly elevated LDH levels in mice. These results suggest that a hypoxia-responsive promoter would facilitate target-specificity and so reduce the side effects on well-oxygenated normal tissues. Consistent with these reports, we did not observe any obvious side effects after the Ad/5HREp-BCD/5-FC gene therapy. On the other hand, after the Ad/EFp-BCD/5-FC treatment, we observed significant weight loss and severe diarrhea, despite the local administration (Figure 5A). These results strengthen further the argument that tumour hypoxia is a specific therapeutic target and our 5HRE system has the advantage of specifically targeting it.

To determine whether the specific targeting of tumour hypoxia by the gene therapy strategy improves the efficacy of radiotherapy in a tumour xenograft, we performed growth delay assays. The gene therapy synergistically kept tumour growth suppressed in combination with the single (12.5 Gy) and the fractionated (3 Gy × 5 fractions) radiotherapy. These results were consistent with the report that a combination of HRE-driven P450R expression and tirapazamine significantly increased the efficacy of radiotherapy *in vivo* (Cowen *et al*, 2004). The data together with ours definitely support that hypoxia-targeting gene therapy combined with radiotherapy is a promising approach to cancer treatment.

Although BCD expression from the *5HREp-BCD* gene was not observed under normoxic conditions in the present Western blotting (Figures 1B and 2B), the cells showed slight but clear sensitivity to a high concentration of 5-FC even under normoxic conditions (Figure 3). This sensitivity was observed regardless of infection with the adenovirus *in vitro* (Figure 3; compare MOI=0–100 in each cell), indicating that an excess dose of 5-FC itself results in BCD-independent cytotoxicity. In our growth delay assays, a significant difference was not observed in TGDT between the IR group and the IR & 5-FC group (without adenovirus administration) (Figure 5B and C and Table 1), indicating that the dose of 5-FC was not excessive, or rather was moderate in our *in vivo* studies. In such an experimental setting, tumour growth in the Ad & 5-FC & IR group was significantly delayed compared to that in the IR group (Figure 5B and C and Table 1). All of these results strongly suggest that the synergistic therapeutic effect of Ad & 5-FC & IR treatment was dependent on the expression of BCD and was caused by the conversion of 5-FC to cytotoxic 5-FU.

Hypoxia-inducible factor-1 plays important roles in regulating tumour radiosensitivity, and therefore, it has been recognised as a potentially promising target for tumour radiosensitisation (Moeller *et al*, 2004; Moeller and Dewhirst, 2006). Because transcription from 5HREp mainly depends on HIF-1 activity, BCD expression from Ad/5HREp-BCD should be induced in the cells with increased HIF-1 activity. In this regard, the gene therapy should have targeted the tumour cells with increased HIF-1 activity and enhanced the therapeutic effect of radiotherapy.

We previously used 5HREp to image hypoxic cells in tumour xenografts (Harada *et al*, 2005; Liu *et al*, 2005). These studies were conducted using tumour xenografts, in which a hypoxia-responsive gene, such as the *5HREp-luciferase* or the *5HREp–GFP* gene, had been previously set, but never using vectors responsible for the *trans*-gene delivery. In the present immunohistochemical analysis, we confirmed the intratumoral expression of BCD after the direct administration of the adenoviral vector into tumour xenografts. The expression was limited to and near the pimonidazole-positive hypoxic regions. Moreover, the BCD was biologically active and indeed led to the antitumour effect we desired. These results represent great progress toward the clinical use of this hypoxia-targeting strategy. However, the most important problem still remains; after the systemic intravenous administration of Ad/5HREp-BCD, we did not detect the expression of BCD in the tumour xenografts in the immunohistochemical analysis (data not shown). For the clinical application of the present gene therapy strategy, the development of a novel gene delivery technology is the next issue to be addressed, although work on this has met with minimal success.

## Figures and Tables

**Table 1:** Statistical analysis of TGDT

	**Single (12.5 Gy)**	**Fractionated (3 Gy × 5)**
Sham-treatment	8.2±3.1	9.8±5.8
Ad & 5-FC	13.2±5.6 (*P*=0.144 *vs* Sham)	13.0±4.4 (*P*=0.148 *vs* Sham)
IR	19.4±4.8	17.0±3.7
IR & 5-FC	19.8±3.4 (*P*=0.865 *vs* IR)	16.8±2.5 (*P*=0.644 *vs* IR)
Ad & 5-FC & IR	47.2±16.8 (*P*<0.01 *vs* IR)	43.3±23.8 (*P*<0.05 *vs* IR)

Abbreviations: 5-FC=5-fluorocytosine; TGDT=tumour growth doubling time.

TGDT was calculated as the mean of the days, on which relative tumor volume of each tumor reached 2-fold of that on day 0. Data were based on the results of the growth delay assays in Figures 5B and C with single (12.5 Gy) and fractionated (3 Gy × 5) irradiation, respectively. Results are the mean of the days±s.d. (*n*=6).

## References

[bib1] Binley K, Askham Z, Martin L, Spearman H, Day D, Kingsman S, Naylor S (2003) Hypoxia-mediated tumour targeting. Gene Therapy 10: 540–549 12646859 10.1038/sj.gt.3301944

[bib2] Bouvet M, Bold RJ, Lee J, Evans DB, Abbruzzese JL, Chiao PJ, McConkey DJ, Chandra J, Chada S, Fang B, Roth JA (1998) Adenovirus-mediated wild-type p53 tumor suppressor gene therapy induces apoptosis and suppresses growth of human pancreatic cancer. Ann Surg Oncol 5: 681–688 9869513 10.1007/BF02303477

[bib3] Brown JM, Wilson WR (2004) Exploiting tumour hypoxia in cancer treatment. Nat Rev Cancer 4: 437–447 15170446 10.1038/nrc1367

[bib4] Chen CA, Okayama H (1987) High-efficiency transformation of mammalian cells by plasmid DNA. Mol Cell Biol 7: 2745–2752 3670292 10.1128/mcb.7.8.2745-2752.1987 PMC367891

[bib5] Chen CA, Okayama H (1988) Calcium phosphate-mediated gene transfer: a highly efficient transfection system for stably transforming cells with plasmid DNA. Biotechniques 6: 632–638 3273409

[bib6] Cowen RL, Williams KJ, Chinje EC, Jaffar M, Sheppard FC, Telfer BA, Wind NS, Stratford IJ (2004) Hypoxia targeted gene therapy to increase the efficacy of tirapazamine as an adjuvant to radiotherapy: reversing tumor radioresistance and effecting cure. Cancer Res 64: 1396–1402 14973055 10.1158/0008-5472.can-03-2698

[bib7] Durand RE, Raleigh JA (1998) Identification of nonproliferating but viable hypoxic tumor cells *in vivo*. Cancer Res 58: 3547–3550 9721858

[bib8] Forsythe JA, Jiang BH, Iyer NV, Agani F, Leung SW, Koos RD, Semenza GL (1996) Activation of vascular endothelial growth factor gene transcription by hypoxia-inducible factor 1. Mol Cell Biol 16: 4604–4613 8756616 10.1128/mcb.16.9.4604 PMC231459

[bib9] Graeber TG, Osmanian C, Jacks T, Housman DE, Koch CJ, Lowe SW, Giaccia AJ (1996) Hypoxia-mediated selection of cells with diminished apoptotic potential in solid tumours. Nature 379: 88–91 8538748 10.1038/379088a0

[bib10] Greco O, Patterson AV, Dachs GU (2000) Can gene therapy overcome the problem of hypoxia in radiotherapy? J Radiat Res (Tokyo) 41: 201–212 11210824 10.1269/jrr.41.201

[bib11] Harada H, Kizaka-Kondoh S, Hiraoka M (2005) Optical imaging of tumor hypoxia and evaluation of efficacy of a hypoxia-targeting drug in living animals. Mol Imaging 4: 182–193 16194450 10.1162/15353500200505112

[bib12] Harris AL (2002) Hypoxia – a key regulatory factor in tumour growth. Nat Rev Cancer 2: 38–47 11902584 10.1038/nrc704

[bib13] Jaakkola P, Mole DR, Tian YM, Wilson MI, Gielbert J, Gaskell SJ, Kriegsheim A, Hebestreit HF, Mukherji M, Schofield CJ, Maxwell PH, Pugh CW, Ratcliffe PJ (2001) Targeting of HIF-alpha to the von Hippel-Lindau ubiquitylation complex by O2-regulated prolyl hydroxylation. Science 292: 468–472 11292861 10.1126/science.1059796

[bib14] Kallio PJ, Pongratz I, Gradin K, McGuire J, Poellinger L (1997) Activation of hypoxia-inducible factor 1alpha: posttranscriptional regulation and conformational change by recruitment of the Arnt transcription factor. Proc Natl Acad Sci USA 94: 5667–5672 9159130 10.1073/pnas.94.11.5667 PMC20836

[bib15] Koshikawa N, Takenaga K, Tagawa M, Sakiyama S (2000) Therapeutic efficacy of the suicide gene driven by the promoter of vascular endothelial growth factor gene against hypoxic tumor cells. Cancer Res 60: 2936–2941 10850440

[bib16] Liu J, Qu R, Ogura M, Shibata T, Harada H, Hiraoka M (2005) Real-time imaging of hypoxia-inducible factor-1 activity in tumor xenografts. J Radiat Res (Tokyo) 46: 93–102 15802864 10.1269/jrr.46.93

[bib17] Miller CR, Williams CR, Buchsbaum DJ, Gillespie GY (2002) Intratumoral 5-fluorouracil produced by cytosine deaminase/5-fluorocytosine gene therapy is effective for experimental human glioblastomas. Cancer Res 62: 773–780 11830532

[bib18] Miyake S, Makimura M, Kanegae Y, Harada S, Sato Y, Takamori K, Tokuda C, Saito I (1996) Efficient generation of recombinant adenoviruses using adenovirus DNA-terminal protein complex and a cosmid bearing the full-length virus genome. Proc Natl Acad Sci USA 93: 1320–1324 8577762 10.1073/pnas.93.3.1320 PMC40078

[bib19] Moeller BJ, Cao Y, Li CY, Dewhirst MW (2004) Radiation activates HIF-1 to regulate vascular radiosensitivity in tumors: role of reoxygenation, free radicals, and stress granules. Cancer Cell 5: 429–441 15144951 10.1016/s1535-6108(04)00115-1

[bib20] Moeller BJ, Dewhirst MW (2006) HIF-1 and tumour radiosensitivity. Br J Cancer 95: 1–5 16735998 10.1038/sj.bjc.6603201 PMC2360497

[bib21] Mullen CA, Kilstrup M, Blaese RM (1992) Transfer of the bacterial gene for cytosine deaminase to mammalian cells confers lethal sensitivity to 5-fluorocytosine: a negative selection system. Proc Natl Acad Sci USA 89: 33–37 1729703 10.1073/pnas.89.1.33 PMC48169

[bib22] Norris ML, Millhorn DE (1995) Hypoxia-induced protein binding to O2-responsive sequences on the tyrosine hydroxylase gene. J Biol Chem 270: 23774–23779 7559551 10.1074/jbc.270.40.23774

[bib23] Ogura M, Shibata T, Yi J, Liu J, Qu R, Harada H, Hiraoka M (2005) A tumor-specific gene therapy strategy targeting dysregulation of the VHL/HIF pathway in renal cell carcinomas. Cancer Sci 96: 288–294 15904470 10.1111/j.1349-7006.2005.00044.x PMC11159877

[bib24] Patterson AV, Williams KJ, Cowen RL, Jaffar M, Telfer BA, Saunders M, Airley R, Honess D, van der Kogel AJ, Wolf CR, Stratford IJ (2002) Oxygen-sensitive enzyme-prodrug gene therapy for the eradication of radiation-resistant solid tumours. Gene Therapy 9: 946–954 12085243 10.1038/sj.gt.3301702

[bib25] Pennacchietti S, Michieli P, Galluzzo M, Mazzone M, Giordano S, Comoglio PM (2003) Hypoxia promotes invasive growth by transcriptional activation of the met protooncogene. Cancer Cell 3: 347–361 12726861 10.1016/s1535-6108(03)00085-0

[bib26] Powis G, Kirkpatrick L (2004) Hypoxia inducible factor-1alpha as a cancer drug target. Mol Cancer Ther 3: 647–654 15141023

[bib27] Rofstad EK (2000) Microenvironment-induced cancer metastasis. Int J Radiat Biol 76: 589–605 10866281 10.1080/095530000138259

[bib28] Semenza GL (2001) HIF-1, O(2), and the 3 PHDs: how animal cells signal hypoxia to the nucleus. Cell 107: 1–3 11595178 10.1016/s0092-8674(01)00518-9

[bib29] Semenza GL (2003) Targeting HIF-1 for cancer therapy. Nat Rev Cancer 3: 721–732 13130303 10.1038/nrc1187

[bib30] Shibata T, Akiyama N, Noda M, Sasai K, Hiraoka M (1998) Enhancement of gene expression under hypoxic conditions using fragments of the human vascular endothelial growth factor and the erythropoietin genes. Int J Radiat Oncol Biol Phys 42: 913–916 9845121 10.1016/s0360-3016(98)00298-3

[bib31] Shibata T, Giaccia AJ, Brown JM (2000) Development of a hypoxia-responsive vector for tumor-specific gene therapy. Gene Therapy 7: 493–498 10757022 10.1038/sj.gt.3301124

[bib32] Shibata T, Giaccia AJ, Brown JM (2002) Hypoxia-inducible regulation of a prodrug-activating enzyme for tumor-specific gene therapy. Neoplasia 4: 40–48 11922390 10.1038/sj.neo.7900189 PMC1503309

[bib33] Teicher BA (1994) Hypoxia and drug resistance. Cancer Metastasis Rev 13: 139–168 7923547 10.1007/BF00689633

[bib34] Thomlinson RH, Gray LH (1955) The histological structure of some human lung cancers and the possible implications for radiotherapy. Br J Cancer 9: 539–549 13304213 10.1038/bjc.1955.55 PMC2073776

[bib35] Vaupel P, Kallinowski F, Okunieff P (1989) Blood flow, oxygen and nutrient supply, and metabolic microenvironment of human tumors: a review. Cancer Res 49: 6449–6465 2684393

[bib36] Vordermark D, Shibata T, Brown JM (2001) Green fluorescent protein is a suitable reporter of tumor hypoxia despite an oxygen requirement for chromophore formation. Neoplasia 3: 527–534 11774035 10.1038/sj.neo.7900192 PMC1506559

[bib37] Wang GL, Jiang BH, Rue EA, Semenza GL (1995) Hypoxia-inducible factor 1 is a basic-helix-loop-helix-PAS heterodimer regulated by cellular O2 tension. Proc Natl Acad Sci USA 92: 5510–5514 7539918 10.1073/pnas.92.12.5510 PMC41725

[bib38] Wang GL, Semenza GL (1993) General involvement of hypoxia-inducible factor 1 in transcriptional response to hypoxia. Proc Natl Acad Sci USA 90: 4304–4308 8387214 10.1073/pnas.90.9.4304 PMC46495

