# Supplementary material for: Adenovirus-mediated hypoxia-targeting cytosine deaminase gene therapy enhances radiotherapy in tumour xenografts
Source: Br J Cancer. 2007 May 22;96(12):1871–8. doi: 10.1038/sj.bjc.6603812 (PMC2359966; doi:10.1038/sj.bjc.6603812)
Supplement: Supplementary Figure Legends [file 6603812x1.doc]

**Supplementary Figure S1** Ad/5HREp-BCD-mediated cytotoxicity. **(A)** HT29 and **(B)** CFPAC-1 cells were infected with the adenovirus, Ad/EFp-BCD or Ad/5HREp-BCD, and treated with various concentrations of 5-FC under normoxic (open) or hypoxic (solid) conditions. Cell viability was calculated as the ratio of the absorbance value under each of the conditions against that in medium with 0.1 mg/ml of 5-FC under normoxic conditions. The same experiment was conducted with mock infection. Results are the mean ± SD (n = 3). There is no significant difference in cytotoxicity between normoxic and hypoxic conditions.

**Supplementary Figure S2**

Hypoxia-responsive DsRed2 expression in HT29 and CFPAC-1 cells. HT29 **(A and B)** and CFPAC-1 **(C and D)** cells were transiently transfected with p5HRE-DsRed2, which encodes a DsRed2 gene under the control of a 5HRE promoter. The cells were cultured under normoxic **(A and C)** and hypoxic **(B and D)** conditions for 16 hours, and reoxygenated for 2 hours.
