# Supplementary material for: Adenovirus-mediated hypoxia-targeting cytosine deaminase gene therapy enhances radiotherapy in tumour xenografts
Source: Br J Cancer. 2007 May 22;96(12):1871–8. doi: 10.1038/sj.bjc.6603812 (PMC2359966; doi:10.1038/sj.bjc.6603812)
Supplement: Supplementary Table S1 [file 6603812x2.doc]

**Supplementary Table S1**

Gene transfer efficiency analyzed by chemiluminescent β-galactosidase assay

| **Cell Line** | **RLUs** |
| --- | --- |
| **HeLa**  **MIA PaCa-2**  **WiDr**  **HT29**  **CFPAC-1** | 7.8 × 10^6^ ( ± 6.7 ×10^5^)  7.7 × 10^6^ ( ± 2.9 ×10^5^)  2.6 × 10^6^ ( ± 1.1 ×10^6^)  2.2 × 10^5^ ( ± 4.9 ×10^4^)  3.2 × 10^5^ ( ± 3.3 ×10^4^) |

The β-gal activity in each cell was normalized to the protein concentration in each cell lysate and is given in as relative luminescent units (RLUs). Results are the mean ± SD (n = 3).
