# Supplementary figures and images for: Adenovirus-mediated hypoxia-targeting cytosine deaminase gene therapy enhances radiotherapy in tumour xenografts
Source: Br J Cancer. 2007 May 22;96(12):1871–8. doi: 10.1038/sj.bjc.6603812 (PMC2359966; doi:10.1038/sj.bjc.6603812)

A HT29

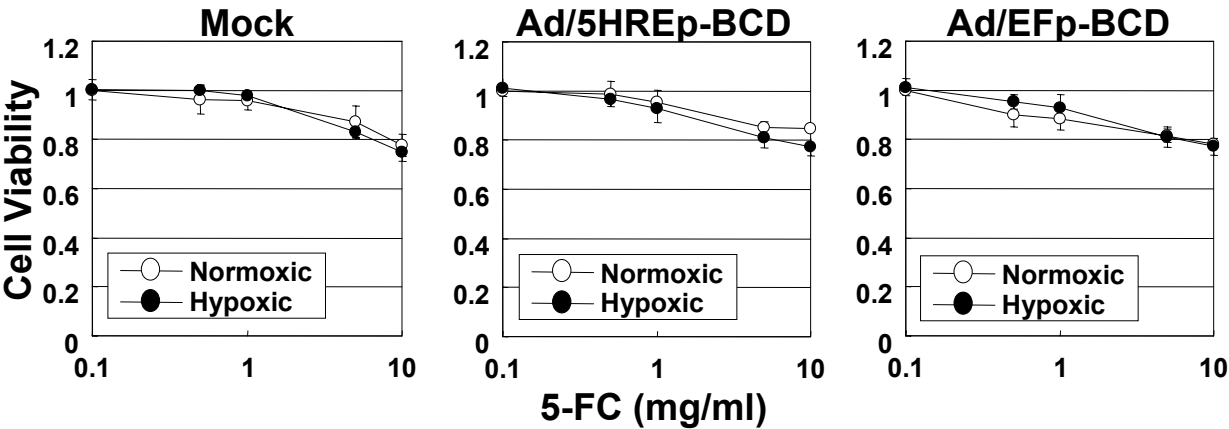

B CFPAC-1

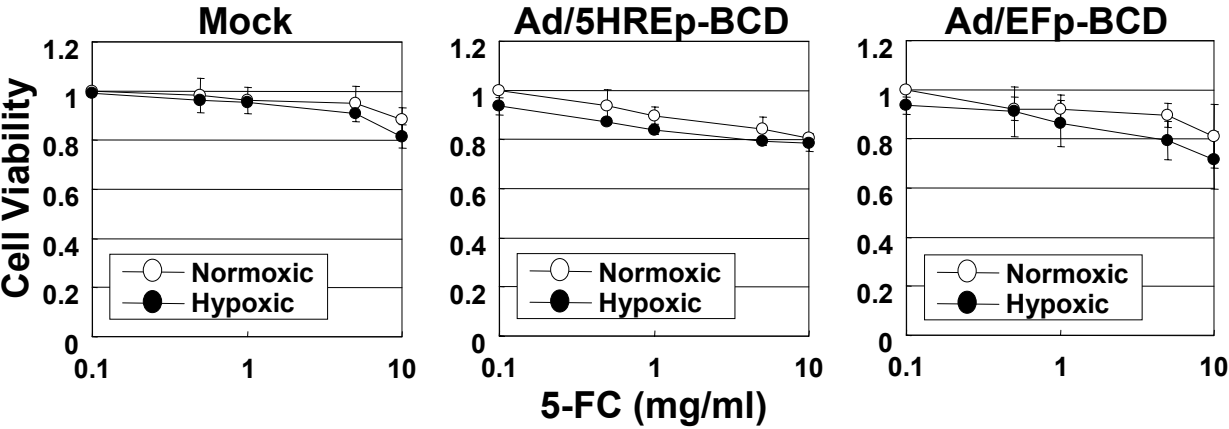

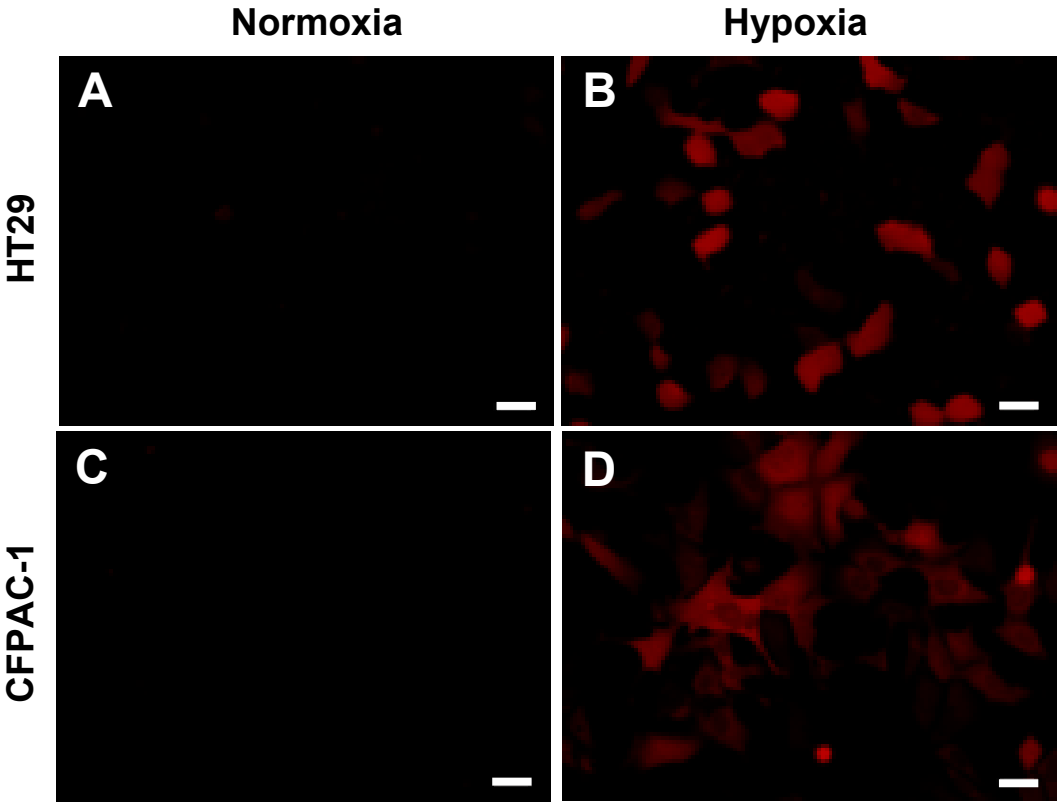

Supplement: Supplementary Figures [file 6603812x3.pdf]
